# *Has2* Regulates the Development of Ovalbumin-Induced Airway Remodeling and Steroid Insensitivity in Mice

**DOI:** 10.3389/fimmu.2021.770305

**Published:** 2022-01-07

**Authors:** Mingma Thsering Sherpa, Takumi Kiwamoto, Masashi Matsuyama, Yoshiya Tsunoda, Kai Yazaki, Kazufumi Yoshida, Masayuki Nakajima, Yosuke Matsuno, Yuko Morishima, Yukio Ishii, Nobuyuki Hizawa

**Affiliations:** Department of Respiratory Medicine, Faculty of Medicine, University of Tsukuba, Tsukuba, Japan

**Keywords:** ER stress response, HAS2, IL-17, TGF-β1, airway remodeling, asthma, mouse model

## Abstract

*HAS2* is a member of the gene family encoding the hyaluronan synthase 2, which can generate high-molecular-weight hyaluronan (HMW-HA). Our previous study identified *HAS2* as a candidate gene for increased susceptibility to adult asthma. However, whether *HAS2* dysfunction affects airway remodeling and steroid insensitivity is still limited. Therefore, this study aimed to clarify the *Has2* dysfunction, triggering severe airway remodeling and steroid insensitivity in a murine model of asthma. *Has2* heterozygous-deficient (*Has2* ^+/−^) mice and their wild-type littermates have been evaluated in a model of chronic ovalbumin (OVA) sensitization and challenge. Mice present a higher sensitivity to OVA and higher IL-17 release as well as eosinophilic infiltration. RNA sequencing demonstrated the downregulation of EIF2 signaling pathways, TGF-β signaling pathways, and heat shock proteins with Th17 bias in *Has2* ^+/−^-OVA mice. The combined treatment with anti-IL-17A antibody and dexamethasone reduces steroid insensitivity in *Has2* ^+/−^-OVA mice. *Has2* attenuation worsens eosinophilic airway inflammation, airway remodeling, and steroid insensitivity. These data highlight that HAS2 and HMW-HA are important for controlling intractable eosinophilic airway inflammation and remodeling and could potentially be exploited for their therapeutic benefits in patients with asthma.

## Introduction

Airway remodeling is an important feature of asthma characterized by goblet cell hyperplasia, subepithelial collagen, and smooth muscle hyperplasia and is known to play a role in persistent airflow obstruction (1). Because airway remodeling is minimally affected by current treatments, prevention of accelerated airway remodeling is one of the important therapeutic targets of asthma (2). Our previous genome-wide association study reported that hyaluronan synthase 2 gene (*HAS2*) is a novel candidate gene for susceptibility to adult asthma (3). Hyaluronan (HA) is an integral component of the extracellular matrix (4). HA synthases synthesize large HA polymers of various sizes. HAS1 and HAS2 produce HA of larger molecular size (2 × 10^4^ kDa), whereas HAS3 synthesizes small-sized HA (2 × 10^2^ kDa) (4). Because high-molecular-weight hyaluronan (HMW-HA) is thought to have an anti-inflammatory function, HAS2 dysfunction was thought to exacerbate asthma. Based on these backgrounds, mouse HAS2 gene (*Has2*) attenuation was recently reported to worsen acute eosinophilic airway inflammation and increase airway hyperresponsiveness (AHR) using *Has2* heterozygous-deficient (*Has2* ^+/−^) mice (5). However, the severity of chronic eosinophilic airway inflammation, airway remodeling, and underlying pathogenesis in *Has2* ^+/−^ mice remains unclear. In this study, the development of airway remodeling after repeated allergen challenges in *Has2* ^+/−^ mice was analyzed to clarify the role of *Has2* in the pathogenesis of airway remodeling in asthma. *Has2* ^+/−^ mice exhibit a more intense allergic eosinophilic airway inflammatory reaction, severe goblet cell hyperplasia, and increased IL-17 level with steroid insensitivity. IL-17 cytokines have been implicated in asthma, and recent studies have suggested activation of steroid-resistant IL-17 pathways in severe asthma patients (6–8). This intractable eosinophilic airway inflammation phenotype can be treated by combined treatment with dexamethasone and anti-IL-17A Ab.

## Methods

### Animals

Because *Has2* homozygous deficient mice are embryonic lethal with severe cardiac and vascular abnormality (9), 6- to 8-week-old female Balb/c mice wild type (WT) and *Has2* heterozygous (*Has* ^+/−^) were used in the experiments. Breeding sets of *Has2* ^+/−^ mice (Jackson Laboratory, Bar Harbor, ME, USA) were backcrossed to Balb/c background for at least eight generations (5, 9). All animal studies were approved by the Institutional Review Board of the University of Tsukuba (approval number: 19-159, 20-125, and 21-028).

### Experimental Protocols

**Figure 1:**
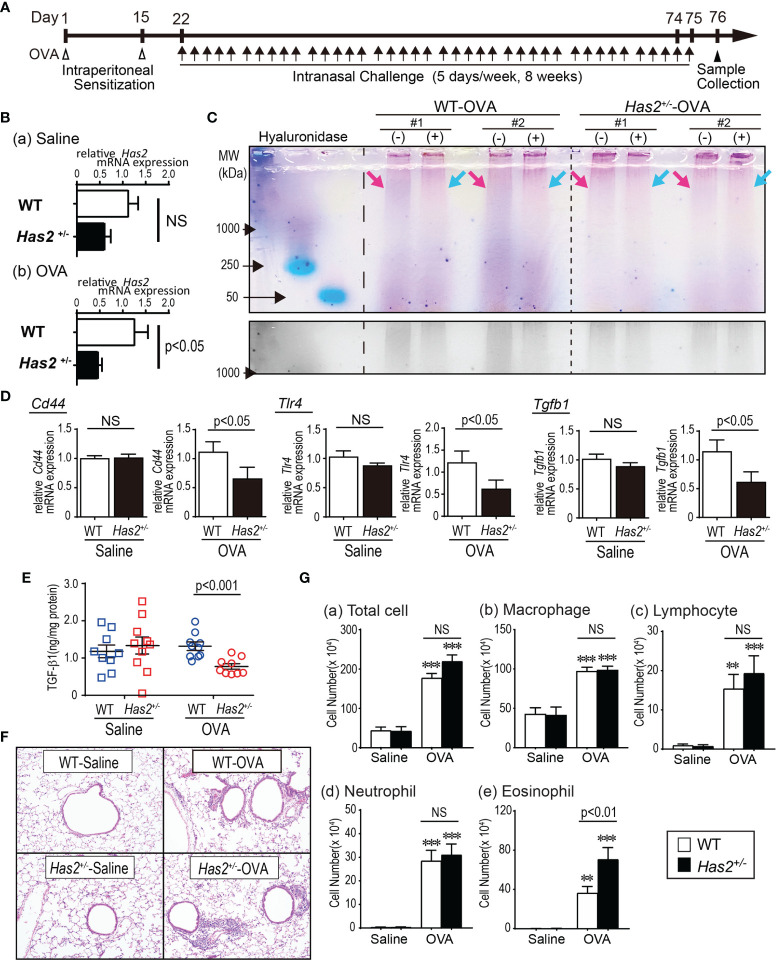
*Has2* attenuation downregulates the HMW-HA, HA-binding protein, and exacerbates airway inflammation in a chronic ovalbumin (OVA)-induced mouse model of asthma. **(A)** Schematic illustration of the experimental design. **(B)** Levels of mRNA transcripts encoding *Has2* (*n* = 6–9). **(C)** (Upper lane) HA size analysis with hyaluronidase treatment. (Lower lane) Grayscale in the high-molecular-weight area. **(D)** Levels of mRNA transcripts encoding *Cd44, Tlr4*, and *Tgfb1* (*n* = 9–10). **(E)** Protein-adjusted levels of TGF-β1 in lung homogenates (*n* = 9–10). **(F)** Lung tissue HE staining. **(G)** BALF cytology of each indicated cell type (*n* = 13–14). All samples are obtained 24 h after the final challenge with saline or OVA. Statistical significance was determined using the Mann–Whitney *U* test **(B, D, E)** or Tukey’s multiple comparison test **(G)**. **P < 0.01, ***P < 0.001 relative to the WT-saline control mice. Horizontal bars indicate direct statistical comparisons between WT and *Has2* ^+/−^ mice. NS, not significant.

Mice were sensitized intraperitoneally with 100 µg of ovalbumin (OVA; Sigma-Aldrich, St. Louis, MO, USA) adsorbed in 1.6 mg of aluminum hydroxide on days 1 and 15. Starting on day 22, mice were challenged intranasally with 10 µg of OVA for 5 days each for eight consecutive weeks (**Figure 1A**). Control mice were injected and challenged with saline. The steroid-treated group received 1 mg/kg dexamethasone (Sigma Aldrich) intraperitoneally into OVA-stimulated WT (WT-OVA) mice and that of *Has2* ^+/−^ (*Has2* ^+/−^-OVA) mice at 24 and 2 h prior to the final intranasal OVA challenge (**Figure 6A**). The combined treatment group received anti-IL-17A monoclonal antibody (100 μg/body, BioLegend, San Diego, CA, USA) intraperitoneally into WT-OVA mice and *Has2* ^+/−^-OVA at 24 and 2 h before final intranasal OVA challenge (**Figure 6A**). Isotype IgG (BioLegend, San Diego, CA, USA) was used as control.

### Quantitative Real-Time RT-PCR

Total RNA was extracted from the snap-frozen lung tissue using a RNeasy^®^ Mini Kit (QIAGEN, Hilden, Germany) according to the protocol of the manufacturer. mRNA expression levels were quantified by the 7500 FAST real-time PCR system (Thermo Fisher Scientific, Waltham, MA, USA). The sequences for the *Has2* ^+/−^ mice-specific TaqMan^®^-MGB probes and primers (Thermo Fisher Scientific) were as follows: Has2F 5′-TGCTTGACCCTGCCTCATC-3′, Has2R 5′-CCCATGAATTCCTGATTGTACCA-3′, MGB probe 5′-TGTCCAGATTTTAAACAAG-3′ (5). Mouse primers and probes were purchased pre-mixed from Thermo Fisher Scientific: CD44 (*Cd44*; Mm01277163_m1), TLR4 (*Tlr4*; Mm00445273_m1), TGF-β1 (*Tgfb1*; Mm01178820_m1), Hsp40 (*Dnajb1*; Mm00444519_m1), Hsp70 (*Hspa1a*; Mm01159846_s1), Herp (*Herpud1*; Mm00445600_m1), PERK (*Eif2ak3*; Mm00438700_m1), ATF4 (*Atf4*; Mm00515325_g1), and GAPDH (*Gapdh*; Mm99999915_g1). All mRNA levels were normalized to *Gapdh* mRNA levels.

### HA Size Analysis

HA size was analyzed as previously described (5, 10). Briefly, right whole lung tissues were digested by incubation in proteinase K (MilliporeSigma, Burlington, MA, USA) at 1 mg/ml in 100 mM ammonium acetate (1 ml/25 mg tissue weight). The samples were then precipitated using ethanol after which nucleic acid digestion followed by second ethanol precipitation was done. After digesting half the samples with hyaluronidase, the samples were resuspended in formamide and then run on agarose gel at a constant voltage. Finally, the gel was stained with Stains-All (Sigma-Aldrich).

### Bronchoalveolar Lavage Fluid Cell Counting

Mouse lungs were lavaged using five repeated instillations of 0.6 ml of saline each through the tracheal cannula. The first 1.2 ml of bronchoalveolar lavage fluid (BALF) was centrifuged, and the supernatant was used for the measurements of various cytokines and chemokines as described (5, 11). Centrifuged cells were redissolved in the remaining BALF sample. Cells were counted using a hemocytometer, and a differential cell count was performed based on count of 300 cells, which morphologically classified the cells based on staining with Diff-Quik (Polysciences, Inc., Warrington, PA, USA), using standard light microscopic techniques.

### Lung Histology

Lung paraffin sections were stained with hematoxylin and eosin (HE) staining to assess airway inflammatory cell infiltration, with periodic acid Schiff (PAS) to demonstrate the presence of mucin within goblet cells, and with Masson’s trichrome (MT) to demonstrate the presence of extracellular matrix. The sections were also stained immunohistochemically using anti-α-smooth muscle actin (α-SMA) antibody (Cell Signaling Technology, Danvers, MA, USA) to identify contractile elements. Morphological analyses of MT and ⍺-SMA-stained regions and %PAS positive cells were performed as described previously (12, 13).

### Airway Hyperresponsiveness to Methacholine

Airway hyperresponsiveness (AHR) to inhaled methacholine (Sigma-Aldrich) in unrestrained mice was measured 24 h after the last intranasal challenge by barometric plethysmography using a whole-body plethysmograph (FinePointe RC System, Buxco, Wilmington, NC, USA). AHR was measured as changes in airway resistance with increasing dose of methacholine (0–50 mg/ml) *via* a jet nebulizer (5).

### Multiplex Cytokine Assay

Cytokines and chemokines in BAL fluids and lung homogenates were measured using MILLIPLEX MAP Kit (MilliporeSigma) according to the instructions of the manufacturer. Twice the recommended sample volume was used for BAL fluid testing as described previously (5).

### Enzyme-Linked Immunosorbent Assay

Quantification of OVA-specific IgE and IgG1 in serum was performed using commercially available ELISA kits (Cayman Chemical, Ann Arbor, MI, USA), whereas TGF-β1 and IL-17F in lung homogenate were measured using ELISA kits (R&D Systems, Minneapolis, MN, USA).

### Lung RNA Extraction and RNA Sequencing

Total RNA was extracted from mouse lungs using the TRIzol^®^ reagent (Thermo Fisher Scientific) according to the instructions of the manufacturer (*n* = 4–5, each group). The concentration and purity of the RNA samples were determined by automated optical density evaluation (OD_260_/OD_280_ ≥ 1.8 and OD_260_/OD_230_ ≥ 1.8) using a NanoDrop spectrophotometer (Thermo Fisher Scientific). RNA sequencing (RNA-seq) libraries were prepared using a NEBNext rRNA Depletion Kit (New England Biolabs, Ipswich, MA, USA) and an ENBNext Ultra Directional RNA Library Prep Kit (New England Biolabs) according to the instructions of the manufacturer using 500 ng of the total RNA samples. Next, 2 × 36 base paired-end sequencing was performed using a NextSeq 500 sequencer (Illumina, San Diego, CA, USA) by Tsukuba i-Laboratory LLP (Tsukuba, Japan). Sequences were mapped to the mm10 mouse genome and quantified using CLC Genomics Workbench version 10.1.1 (QIAGEN). An adjusted *P*-value <0.01 (Benjamini–Hochberg FDR method for multiple testing corrections) and relative changes in transcription levels >1.2-fold were used as the cutoff criterion (**Supplemental Figure 1**). The data are available under GEO series accession number GSE181966.

### Pathway Analysis of Differentially Expressed Genes and CIBERSORT Analysis

Identification of the unique differentially expressed genes (DEGs) between WT-saline vs. WT-OVA and WT-saline vs. *Has2* ^+/−^-OVA was done by using Venny (v2.0; http://bioinfogp.cnb.csic.es/tools/venny/index.html). Biological pathways enriched in the data were identified with Ingenuity Pathway Analysis (IPA) software (QIAGEN) using Fisher’s exact test (*P* < 0.05 indicates statistical significance). CIBERSORT analysis was performed on RNA-seq data by using the analytical tool (https://cibersort.stanford.edu/) (14). Previously published mouse reference signature matrix, consisting of 511 distinguishing genes for 25 immune cell types, was used as the reference profile (15).

### Statistical Analysis

Data are shown as means ± SEMs or individual dot plots with means ± SEMs. Statistical significance between groups was evaluated using Mann–Whitney *U* test or ANOVA with Tukey’s multiple comparison test. *P*-values <0.05 were considered statistically significant.

## Results

### The Expression Level of *Has2* mRNA and HMW-HA Is Reduced in Lung Tissues of OVA-Challenged *Has2^+/−^* Mice

To establish the role of *Has2* in chronic allergic airway inflammation, mice were intranasally stimulated with OVA for 8 weeks (**Figure 1A**). *Has2* mRNA expression was significantly lower in the OVA-stimulated *Has2* ^+/−^ (*Has2* ^+/−^-OVA) mice than those in the OVA-stimulated WT (WT-OVA) mice (**Figure 1B**). The genetic modulation of *Has2* did not affect the expression of *Has2* mRNA in the saline control group (**Figure 1B**). Next, to elucidate whether *Has2* mRNA attenuation affects the HMW-HA levels, HA size analysis was performed. Hyaluronidase-sensitive HMW-HA band was less abundant in the lung extracts of *Has2* ^+/−^-OVA mice than in the lung tissues of WT-OVA mice (**Figure 1C**). These results supported the hypothesis that the expression level of *Has2* mRNA in *Has2* ^+/−^ mice was downregulated during chronic eosinophilic airway inflammation, and *Has2* dysfunction impaired the HMW-HA production.

### *Has2* Attenuation Results in the Downregulation of HA-Binding Proteins and TGF-β1

CD44 and TLR4 are known as HA-binding proteins and decreased CD44 downregulates the TGF-β (16). To determine whether *Has2* mRNA attenuation affects the expression of HA-binding protein and downstream molecules, mRNA expression levels of *Cd44*, *Tlr4*, and *Tgfb1* were evaluated. After the OVA stimulation, expression levels of *Cd44*, *Tlr4*, and *Tgfb1* mRNA were significantly lower in the lungs from *Has2* ^+/−^-OVA mice than those from WT-OVA mice (**Figure 1D**). TGF-β1 levels in the lung homogenate were significantly lower in *Has2* ^+/−^-OVA mice than those in WT-OVA mice (**Figure 1E**). These results supported the hypothesis that *Has2* attenuation impaired the expression of HA-binding protein and TGF-β signaling.

### *Has2* Attenuation Enhances OVA-Induced Eosinophilic Airway Inflammation in Mice

Increased inflammatory cells were demonstrated after a repeated OVA exposure, especially in the peribronchial and perivascular areas of both *Has2* ^+/−^ mice and WT mice (**Figure 1F**). To clarify the roles of *Has2* attenuation in the development of OVA-induced chronic airway inflammation, the number of inflammatory cells in BALF was determined (**Figure 1G**). The number of eosinophils was significantly higher in *Has2* ^+/−^-OVA mice than that in WT-OVA mice (**Figure 1G**). These results indicate that *Has2* attenuation worsens the OVA-induced chronic airway inflammation.

### Airway Goblet Cell Hyperplasia Is Significantly Increased in Chronic OVA-Stimulated *Has2^+/−^* Mice

**Figure 2:**
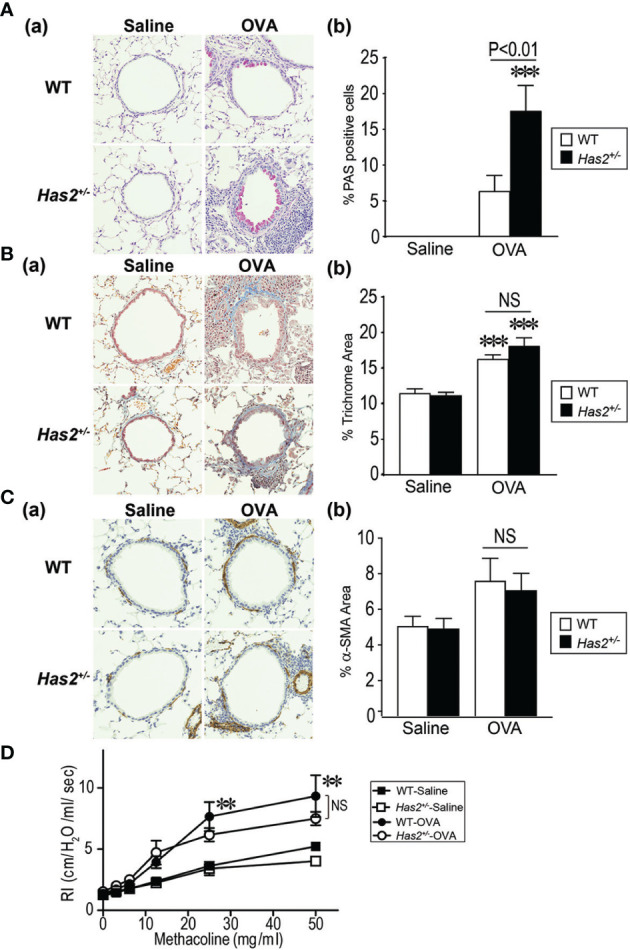
Airway remodeling phenotype of *Has2* ^+/−^ mice after chronic OVA stimulation. **(A)** Periodic acid Schiff (PAS) stain in WT and *Has2* ^+/−^ mice (panel **a**). Percentage of PAS-positive cells in the airway epithelium of WT and *Has2* ^+/−^ mice (panel **b**, *n* = 8–9). **(B)** Masson’s trichrome stain in WT and *Has2* ^+/−^ mice (panel **a**). Percentage of trichrome staining area in the 20-μm region beneath the epithelium in the airways of WT and *Has2* ^+/−^ mice (panel **b**, *n* = 8–9). **(C)** Alpha smooth muscle actin (⍺-SMA) stain in WT and *Has2* ^+/−^ mice (panel **a**). Percentage of ⍺-SMA staining area in the 20-μm region beneath the epithelium in the airways of WT and *Has2* ^+/−^ mice (panel **b**, *n* = 5). **(D)** Airway hyperresponsiveness to methacholine in WT and *Has2* ^+/−^ mice (*n* = 5–9). All samples are obtained 24 h after the final challenge with saline or OVA. Statistical significance is determined using Tukey’s multiple comparison test (**A–C**, panel **b**) or two-way ANOVA **(D)**. **P < 0.01, ***P < 0.001 relative to the WT-saline mice. Horizontal bars indicate direct statistical comparisons between WT-OVA and *Has2* ^+/−^-OVA mice. NS, not significant.

We next evaluated the role of *Has2* in goblet cell hyperplasia, one of the characteristic features of airway remodeling, in WT and *Has2* ^+/−^ mice after repeated challenges with OVA or saline. Only a few epithelial cells were positive for PAS staining in the airway of both mouse genotypes after the saline challenge (**Figure 2A, panel a**). Morphometric analysis showed that %PAS-positive mucus production cells were significantly higher in *Has2* ^+/−^-OVA mice airways than in WT-OVA mice airways (**Figure 2A, panel b**). These results indicate that *Has2* attenuation induces goblet cell hyperplasia and mucus hyperproduction.

### Parameters of Airway Fibrosis, Airway Smooth Muscle Hyperplasia, and AHR Are not Significantly Different Between OVA-Stimulated WT and *Has2^+/−^* Mice

To further evaluate the characteristic features of airway remodeling, mice lung sections were stained with MT staining or immunohistochemically stained with anti-α-SMA antibody (**Figures 2B, C**). Substantial subepithelial deposition of extracellular matrix was strongly observed after repeated OVA challenges in MT-stained lungs of both WT and *Has2* ^+/−^ mice (**Figure 2B, panel a**). Morphometric analysis revealed that the area percentage of extracellular matrix in the 20-mm region beneath the epithelium was significantly higher after OVA challenges as compared with saline challenges in both mouse genotypes (**Figure 2B, panel b**). However, increased airway fibrosis was not significantly different between the WT-OVA and *Has2* ^+/−^-OVA groups. We next evaluated the degree of smooth muscle cell hypertrophy in the airways of both WT and *Has2* ^+/−^ mice. Although thin smooth muscle cell layer that was stained positive for anti-α-SMA antibody was observed in the airways of both genotypes (**Figure 2C, panel a**), the levels of airway smooth muscle hyperplasia were not significantly different between the WT-OVA and *Has2* ^+/−^-OVA groups (**Figure 2C, panel b**). Furthermore, to determine whether the attenuation of *Has2* worsens airway hyperreactivity in chronic OVA-stimulated conditions, airway resistance (RI) with an increasing dose of methacholine was measured. Although RI values were higher in both WT-OVA and *Has2* ^+/−^-OVA mice than the WT-saline mice in a methacholine dose-dependent manner, RI values were not significantly different between the WT-OVA and *Has2* ^+/−^-OVA mice (**Figure 2D**).

### OVA-Stimulated *Has2^+/−^* Mice Demonstrate Increased IL-17 Level

**Figure 3:**
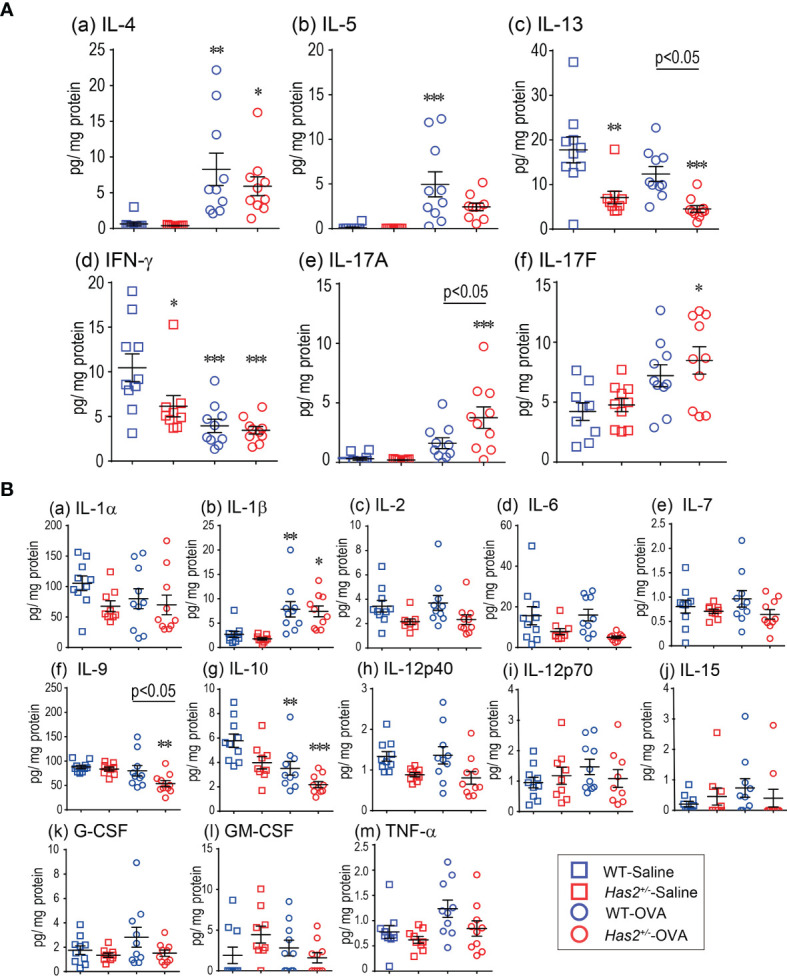
Effects of *Has2* attenuation on various cytokine and chemokine levels in lung homogenate. **(A)** Protein-adjusted levels of the indicated Th1/Th2- and Th17-related cytokines in lung homogenates (*n* = 9–10). **(B)** Protein-adjusted levels of inflammatory cytokines and chemokines in lung homogenates (*n* = 9–10). All samples are obtained 24 h after the final challenge with saline or OVA. Statistical significance was determined using Tukey’s multiple comparison test. *P < 0.05, **P < 0.01, and ***P < 0.001 relative to WT-saline mice. Horizontal bars indicate direct statistical comparisons between WT-OVA and *Has2* ^+/−^-OVA mice.

To test the hypothesis that eosinophilic airway inflammation increases in *Has2* ^+/−^ mice due to altered allergic cytokine and chemokine responses, the levels of several inflammatory mediators were measured in lung homogenate and BALF samples (**Figure 3** and **Supplemental Figures 2, 3**). IL-17A levels in lung homogenate were significantly higher in *Has2* ^+/−^-OVA mice than that in WT-OVA mice (**Figure 3A, panel e**). ELISA assay also revealed that IL-17F was significantly higher in *Has2* ^+/−^-OVA mice than that in WT-OVA mice (**Figure 3A, panel f**). Interestingly, IL-9 and IL-13 were significantly lower in *Has2* ^+/−^-OVA mice than those in WT-OVA mice (**Figures 3A, panels c, b, f**). In BALF samples, only MCP-1 and RANTES were significantly higher in *Has2* ^+/−^-OVA mice than those in WT-OVA mice (**Supplemental Figure 3**). These results indicate *Has2* attenuation induced severe eosinophilic airway inflammation and mucus hypersecretion due to increased IL-17.

### Attenuation of *Has2* Does not Affect Serum Immunoglobulin Levels in Chronic OVA-Stimulated Condition

To examine whether the *Has2* ^+/−^ mice displayed any other evidence of enhanced systemic allergic response, serum OVA-specific IgE and IgG1 were measured. Chronic OVA challenge significantly increased the serum OVA-specific IgE and IgG1 levels in both *Has2* ^+/−^ and WT mice as compared with the saline control mice, respectively (**Supplemental Figure 4**). When the levels of these antibodies were directly compared between WT-OVA and *Has2* ^+/−^-OVA mice, no significant differences were observed (**Supplemental Figure 4**), indicating that *Has2* attenuation did not cause systemic allergic responses.

### Attenuation of *Has2* Impairs TGF-β and Endoplasmic Reticulum Stress Response-Related Signaling

**Figure 4:**
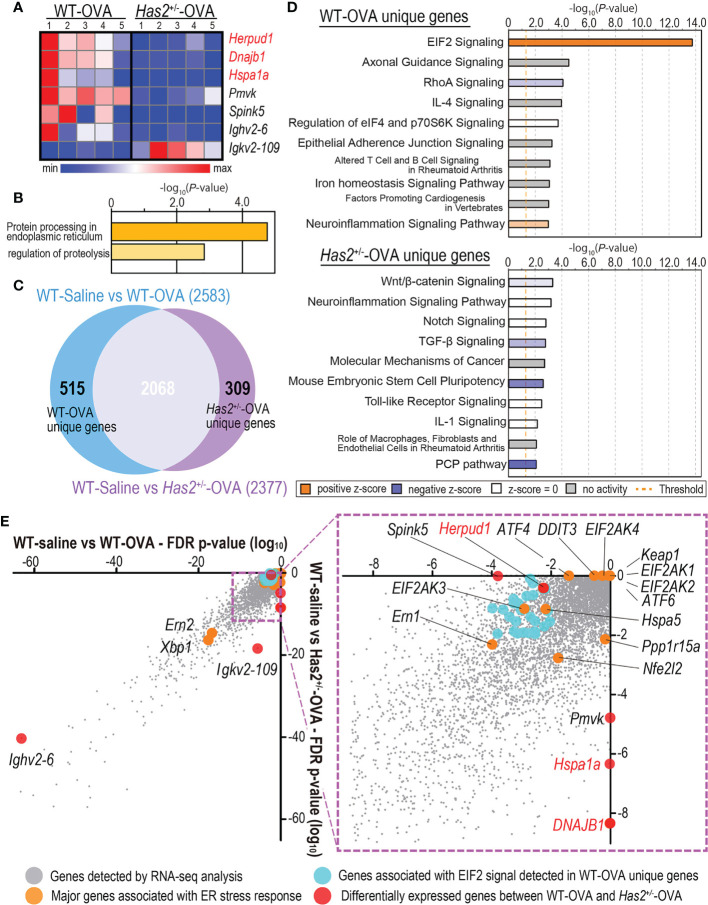
DEG analysis between the lungs from WT-saline, WT-OVA, and *Has2* ^+/−^-OVA mice. **(A)** Heatmap of DEGs between the lungs of WT-OVA and *Has2* ^+/−^-OVA mice (*n* = 5, cutoff: adjusted *P* < 0.01; log_2_ fold change > 1.2). Hierarchical clustering was performed based on the mean log_2_ fold change. Gene symbols in red are associated with endoplasmic reticulum stress response. **(B)** Significant biological process terms detected by GO analysis from WT-OVA and *Has2* ^+/−^-OVA mice. **(C)** Identification of the unique DEGs between WT-saline vs. WT-OVA and WT-saline vs. *Has2* ^+/−^-OVA using the Venn diagram. **(D)** Top 10 significant terms detected by pathway analysis in WT-OVA unique (upper panel) and *Has2* ^+/−^-OVA unique genes (lower panel). **(E)** DEG *P*-value correlation plot between WT-saline vs. WT-OVA and WT-saline vs. *Has2* ^+/−^-OVA. DEGs, differentially expressed genes.

**Figure 5:**
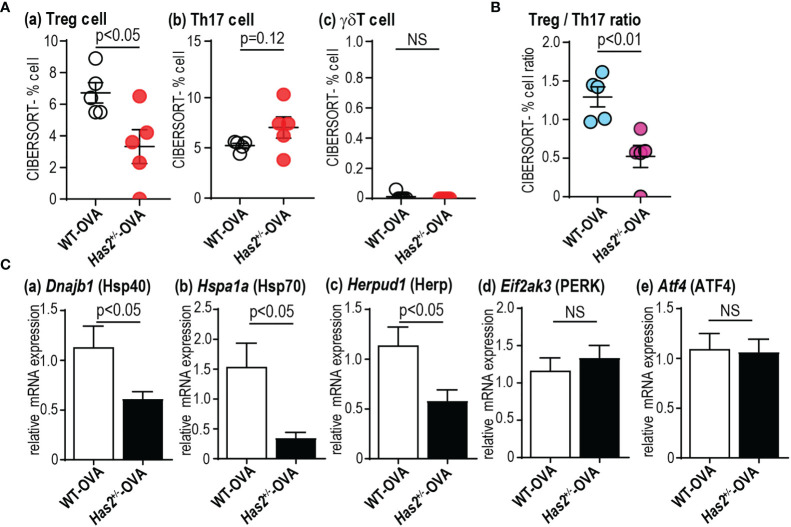
*Has2* attenuation induces Th17/Treg imbalance and attenuation of ER stress response-related molecules. Semiquantitative evaluation of immune cell infiltrates **(A)** and Treg/Th17 ratio **(B)** after the OVA stimulation as determined by CIBERSORT analysis on whole-lung RNA-seq data (*n* = 5). **(C)** Levels of mRNA transcripts encoding *Dnajb1*, *Hspa1a*, *Herpud1*, *Eif2ak3*, and *Atf4* (*n* = 9–10). All samples are obtained 24 h after the final challenge with OVA. Statistical significance was determined using the Mann–Whitney *U* test. Horizontal bars indicate direct statistical comparisons between WT and *Has2* ^+/−^ mice. NS, not significant.

To understand gene expression changes after a chronic OVA challenge in WT and *Has2* ^+/−^ mice, RNA-seq was performed. Seven genes were found to have significantly different expressions between the lungs of WT-OVA mice and *Has2* ^+/−^-OVA mice (**Figure 4A**). Gene ontology analyses identified a significant biological process, such as “protein processing in the endoplasmic reticulum (ER)” and “regulation of proteolysis” by three downregulated genes (**Figure 4B**). Although a significant attenuation of *Spink5* was thought to be involved in childhood asthma through the interaction with TSLP in *Has2* ^+/−^-OVA mice (17), the expression levels of *Tslp* were not significantly different between WT-OVA and *Has2* ^+/−^-OVA mice. Next, we focused our analysis on specifically altered genes in WT-OVA and *Has2* ^+/−^-OVA mice as compared with WT-saline mice (**Figure 4C**). A total of 515 genes were uniquely altered in WT-OVA mice (WT-OVA unique genes, **Figure 4C**), whereas 307 genes were uniquely altered in *Has2* ^+/−^-OVA mice (*Has2* ^+/−^-OVA unique genes, **Figure 4C**). Pathway analysis revealed that the “EIF2 signaling” pathway was the most significantly activated pathway in WT-OVA unique genes (**Figure 4D**, upper panel, and **Supplemental Table 1**). Conversely, “TGF-β signaling” was significantly inhibited in *Has2* ^+/−^-OVA unique genes. Pathways, such as “Wnt/β-catenin signaling” and “PCP pathway”, were significantly inhibited in both WT-OVA unique genes and *Has2* ^+/−^-OVA unique genes (**Figure 4D**, lower panel, and **Supplemental Table 2**). EIF2 signaling is known as one of the major ER stress responses. A comparison of DEGs revealed that not only EIF2 signaling-related genes in WT-OVA mice but also significant changes of heat shock protein (Hsp)-related genes, such as *Hspa1a* (Hsp70) and *Dnajb1* (Hsp40), were found in the genes of *Has2* ^+/−^-OVA mice (**Figure 4E**). To account for the striking cytokine changes in the lungs between WT-OVA and *Has2* ^+/−^-OVA, CIBERSORT analysis was performed. In this analysis, % Treg cell was significantly decreased in *Has2* ^+/−^-OVA mice (**Figure 5A**). Conversely, we observed an increasing trend of %Th17 population in *Has2* ^+/−^-OVA mice (**Figure 5A**). Thus, the Treg/Th17 ratio significantly decreased in *Has2* ^+/−^-OVA mice after repeated allergen challenges (**Figure 5B**). These results indicate that Th17 bias occurs in *Has2* ^+/−^-OVA under these conditions. Furthermore, quantitative reverse transcription-polymerase chain reaction was performed to confirm these DEG changes. *Hspa1a*, *Dnajb1*, and *Herpud1* mRNA expression levels were significantly lower in the lungs of *Has2* ^+/−^-OVA mice (**Figure 5C**). However, gene expression of *Eif2ak3* and *Atf4*, known to be important in the EIF2 signaling pathway, was not significantly different between the two groups.

### *Has2* Attenuation Induces Steroid Resistance but Anti-IL-17A Ab Effective Intractable Airway Inflammation

**Figure 6:**
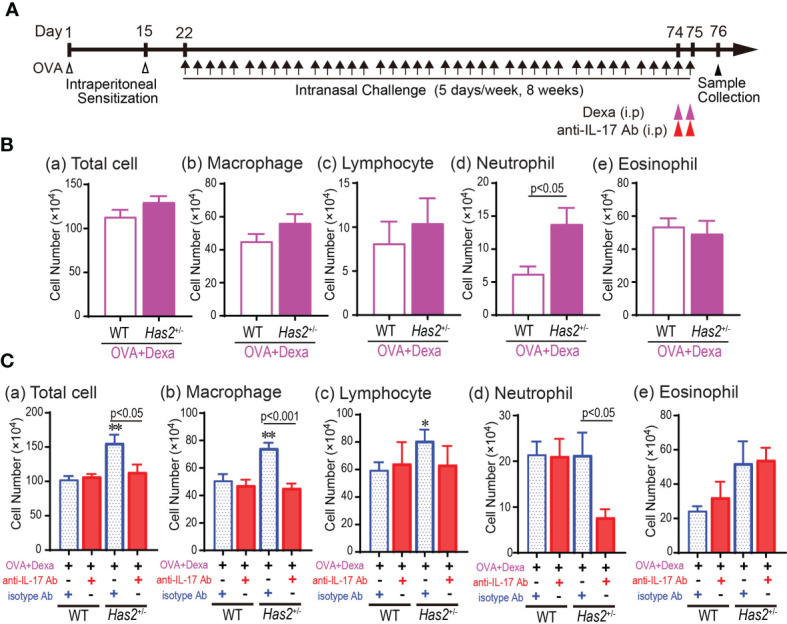
Combined treatment with dexamethasone and anti-IL-17A Ab is effective for steroid-insensitive airway inflammation in *Has2* ^+/−^ mice. **(A)** Schematic illustration of the experimental design for steroid insensitivity and neutralization experiment. **(B)** BALF cytology of each indicated cell type after the dexamethasone treatment from WT-OVA mice and *Has2* ^+/−^-OVA mice (*n* = 8–11). **(C)** BALF cytology of each indicated cell type after a combined treatment with dexamethasone and anti-IL-17A Ab from WT-OVA and *Has2* ^+/−^-OVA mice (*n* = 7–11). All samples are obtained 24 h after the final challenge with OVA. Statistical significance was determined using the Mann–Whitney *U* test. *P < 0.05 and **P < 0.01 relative to WT-OVA-Dexa mice. Horizontal bars indicate direct statistical comparisons between WT-OVA and *Has2* ^+/−^-OVA mice. Dexa, dexamethasone; i.p., intraperitoneal injection; NS, not significant.

Increased IL-17A level is known to mediate the development of neutrophilic airway inflammation *via* corticosteroid resistance. To clarify the effects of *Has2* attenuation on steroid-resistant phenotype in *Has2* ^+/−^ mice, Dexa was administered to both WT-OVA and *Has2* ^+/−^-OVA mice (**Figure 6A**). The number of total cells and macrophages in BALF decreased in both Dexa-treated WT-OVA (WT-OVA-Dexa) and *Has2* ^+/−^-OVA (*Has2* ^+/−^-OVA-Dexa) mice as compared with Dexa-untreated mice (**Figure 6B**). However, a significant increase in the number of neutrophils remained in BALF of *Has2* ^+/−^-OVA-Dexa mice than that of WT-OVA-Dexa mice (**Figure 6B**). These results indicate that *Has2* attenuation induces a steroid-insensitive airway inflammation phenotype. To confirm whether increased IL-17 level induces steroid resistance in phenotypes of *Has2* ^+/−^-OVA-Dexa mice, anti-IL-17A-neutralizing antibodies were administered to both WT-OVA-Dexa and *Has2* ^+/−^-OVA-Dexa mice (**Figure 6A**). Treatment with anti-IL-17A significantly attenuated macrophages and neutrophil counts in the lungs of *Has2* ^+/−^-OVA-Dexa mice than those of isotype control Ab-treated *Has2* ^+/−^-OVA-Dexa mice (**Figure 6C**). Conversely, anti-IL-17A treatment did not demonstrate an additional attenuation of neutrophil counts in the lungs of WT-OVA-Dexa mice than that of isotype control Ab-treated WT-OVA-Dexa mice (**Figure 6C**). Collectively, these results indicate that IL-17 is required to drive steroid resistance intractable to asthma development in *Has2* ^+/−^ mice.

## Discussion

This study found several novel findings. First, the results confirm and extend the findings of Yatagai et al. by showing insufficient mRNA expression of *Has2* in the lungs of *Has2* ^+/−^ mice (**Figure 1B**) and decreased HMW-HA in *Has2* ^+/−^ mice lung after chronic OVA stimulation (**Figure 1C**) (3). Although our previous study on acute OVA stimulation reported a significant decrease of *Has2* mRNA level in the lungs of *Has2* ^+/−^ mice, HMW-HA levels were increased in both WT and *Has2* ^+/−^ mice that might be affected by *Has1* at the early phase of OVA stimulation (5, 18, 19). In this study, a significant attenuation of both *Has2* mRNA expression and HMW-HA levels was observed in the lungs of *Has2^+/−^* mice after a chronic OVA stimulation. This finding was consistent with our hypothesis that *Has2* heterozygous deficiency results in the attenuation of Has2 expression and HMW-HA, which have anti-inflammatory activity, during chronic eosinophilic airway inflammation. CD44 and TLR4 are known as HA-binding proteins, and a decrease of CD44 downregulates TGF-β (16, 20–22). As we previously found, a significant downregulation of *Cd44*, *Tlr4*, and *Tgfb1* mRNA levels was also observed in the lungs of *Has2* ^+/−^-OVA mice (**Figure 1D**) (5). Furthermore, we found a significant decrease of lung TGF-β levels in *Has2* ^+/−^-OVA mice (**Figure 1E**). Collectively, our results suggest that *Has2* gene abnormalities cause downregulation of *Has2* mRNA expression, HMW-HA, and HA-binding protein levels during chronic eosinophilic airway inflammation. Thus, these *Has2* genetic abnormalities impair hyaluronic-acid-induced homeostasis in the asthmatic mouse airway.

Second, this study firstly demonstrated that attenuation of *Has2* mRNA affects the severity of pulmonary eosinophilic inflammation and distinctive phenotype airway remodeling using *Has2^+/−^* mice. *Has2^+/−^* mice showed an increased number of BALF eosinophils after an OVA stimulation (**Figure 1G**). Severe goblet cell hyperplasia was also observed in *Has2^+/−^*-OVA mice (**Figure 2A**). However, no changes in subepithelial fibrosis, airway smooth muscle hypertrophy, and AHR were observed (**Figures 2B**–**D**). In the cytokine and chemokine analyses, not only a significant decrease of TGF-β levels in the *Has2* ^+/−^-OVA mice lung but also increased IL-17 levels were observed (**Figure 3A**). However, no significant increases were observed in Th2-type cytokines and airway epithelium-related cytokine levels in the lung homogenates nor in serum OVA-specific immunoglobulin levels in *Has2^+/−^*-OVA mice (**Figure 3** and **Supplemental Figures 2**–**4**). IL-17 is known to enhance airway eosinophilia, AHR, and mucus hypersecretion (23–25). Furthermore, TGF-β has a protective role for airway inflammation and AHR (26, 27), but worsens the airway fibrosis and airway muscle hyperplasia (28). Collectively, these results suggested that *Has2* attenuation induced distinctive phenotype of OVA-induced airway remodeling by mediating IL-17 and TGF-β signaling.

RNA-seq analysis provided further pathophysiological insights into enhanced eosinophilic airway inflammation when *Has2* expression was attenuated. Although only seven DEGs were identified between WT-OVA and *Has2* ^+/−^-OVA mice, *Dnajb1*, *Hspa1a*, and *Herpud1* were related to the ER stress response and Hsp (**Figures 4A, E**). Pathway analysis revealed that “EIF2 signaling” was the most significant activated pathway in WT-OVA unique genes, whereas “TGF-β signaling” was significantly inhibited in *Has2* ^+/−^-OVA unique genes (**Figure 4D**). EIF2 signaling is one of the major ER stress sensor pathways associated with unfolded protein response (UPR). A previous study reported that EIF2 signaling was downregulated in patients with childhood asthma (29). Moreover, pathways associated with ER stress and UPR play a role in modulating inflammatory and immune responses in the development of severe asthma (30). Furthermore, administration of the ER stress inhibitor decreased the IL-17 expression (31). These results suggest that impaired ER stress response might be implicated in *Has2*-dysfunction-induced asthma. Interestingly, in CIBERSORT analysis, Th17/Treg balance was significantly Th17 biased in *Has2* ^+/−^-OVA mice than that in WT-OVA mice (**Figures 5A, B**). A recent study revealed that TGF-β regulates iTreg and Th17 cell differentiation by both Smad3- and TAK1-dependent pathways in OVA-induced airway inflammation (32). Conversely, evidence suggests the involvement of Hsp, including Hsp70, in the development of psoriasis, characterized by impaired immunological cell function with altered Th17/Treg balance, autoreactive T cells, and dysregulation of keratinocyte proliferation (33, 34). Furthermore, *Dnajb1* controls the substrate targeting HSP70 (35), and *Dnajb1* overexpression downregulates Th17 differentiation in mouse spleen lymphocytes (36). In this study, we found a significant decrease of TGF-β, *Hspa1a*, and *Dnajb1* mRNA expressions in the lungs of *Has2* ^+/−^-OVA mice (**Figure 5C**). These decreases may induce the development of a Th17-dominant phenotype.

Although the role of IL-17 in asthma is unclear, IL-17 is thought to be related to asthma severity (37, 38), exacerbations (39, 40), and steroid insensitivity (6–8, 37, 41), particularly in neutrophilic asthma (6). In our previous acute OVA-induced eosinophilic airway model, we also observed more severe lung neutrophilia with IL-17A signaling modification in OVA-stimulated *Has2^+^* ^/−^ mice (5). However, both neutrophils were similarly elevated in this study. The most likely reason was that both groups were saturated with neutrophil-inducing stimuli due to the daily stimulation of OVA. The present study revealed that OVA-induced airway inflammation in *Has2* ^+/−^-OVA mice was resistant to steroid treatment (**Figure 6B**). Notably, this refractory airway inflammation was relieved by combined therapy with steroid and anti-IL-17A antibodies (**Figure 6C**). Recent literature reported that combined administration of anti-IL-17A Ab and corticosteroid significantly attenuated steroid-insensitive airway inflammation, AHR, and body weight loss (42). Furthermore, Pathinayake et al. reported that heightened ER stress is associated with severe eosinophilic and neutrophilic inflammation in asthma and ER stress genes displayed a significant correlation with classic Th2 genes and Th17 (IL-17F/CXCL1) genes (43). These results indicate that both anti-IL-17A and anti-IL-17F antibodies are potential candidates for the treatment of refractory asthma with ER stress response abnormalities. The reason for the neutrophil increase in WT-OVA mice treated with steroid and isotype control Ab (Rat IgG1, κ) is unknown but could be due to induced non-specific inflammation by the isotype Ab itself.

Several potential shortcomings of the current work should be addressed. For instance, the airway glycan ligand that acts on CD44 or ER stress response may be reduced in *Has2^+/−^* mice. However, this reduction has not been confirmed. The deletion of *Has2* might affect these mice in other ways. Future experiments are needed, for example, to determine whether the *Has2* genetic deletion directly affects inflammatory cells and whether the supply of HAS2 enzyme, HMW-HA, and Hsp into the airway abrogates the lung eosinophilia. Moreover, the evaluation of ER stress and Hsp responses in each tissue and cell was insufficient; therefore, detailed tissue- or cell-specific examination, such as single-cell RNA-seq analysis, will be needed. In addition, to clarify the efficacy of treatment, it is necessary to study the effect of anti-IL-17A antibody alone, but this has not been done.

Nevertheless, the fact that *Has2^+/−^* mice have more severe airway remodeling, steroid insensitivity, and fewer HMW-HA in their airways strongly suggests that reduced levels of *Has2* impair extracellular matrix homeostasis for controlling chronic airway eosinophilia. Furthermore, disorders associated with reduced HAS2 function in the lungs might manifest intractable airway inflammation and remodeling with the Th17 bias. These data also support the notion that HAS2 and HMW-HA are important for controlling steroid-resistant eosinophilic airway inflammation and remodeling and could potentially be exploited for therapeutic benefits.

## Data Availability Statement

The datasets presented in this study can be found in online repositories. The names of the repository/repositories and accession number(s) can be found below: https://www.ncbi.nlm.nih.gov/geo/, GSE181966.

## Ethics Statement

The animal study was reviewed and approved by the Institutional Review Board of the University of Tsukuba.

## Author Contributions

MS, TK, MM, YI, and NH conceived, designed, and performed the experiments. MS, MM, and TK analyzed the data. MS, YT, KYa, KYo, MN, YMa, and YMo contributed the reagents, materials, and/or analysis tools. MS and TK wrote the manuscript. All authors contributed to the article and approved the submitted version.

## Funding

This research was supported in part by grants-in-aid from the Ministry of Education, Culture, Sports, Science, and Technology of Japan (18K08139 and 21K08150 to TK) and the Basic Research Support Program from Japanese Society of Allergology (to TK).

## Conflict of Interest

The authors declare that the research was conducted in the absence of any commercial or financial relationships that could be construed as a potential conflict of interest.

## Publisher’s Note

All claims expressed in this article are solely those of the authors and do not necessarily represent those of their affiliated organizations, or those of the publisher, the editors and the reviewers. Any product that may be evaluated in this article, or claim that may be made by its manufacturer, is not guaranteed or endorsed by the publisher.
