# Supplementary material for: Has2 Regulates the Development of Ovalbumin-Induced Airway Remodeling and Steroid Insensitivity in Mice
Source: Front Immunol. 2022 Jan 7;12:770305. doi: 10.3389/fimmu.2021.770305 (PMC8777110; doi:10.3389/fimmu.2021.770305)
Supplement: Supplementary file 1 [file DataSheet_1.pdf]

Supplementary Material

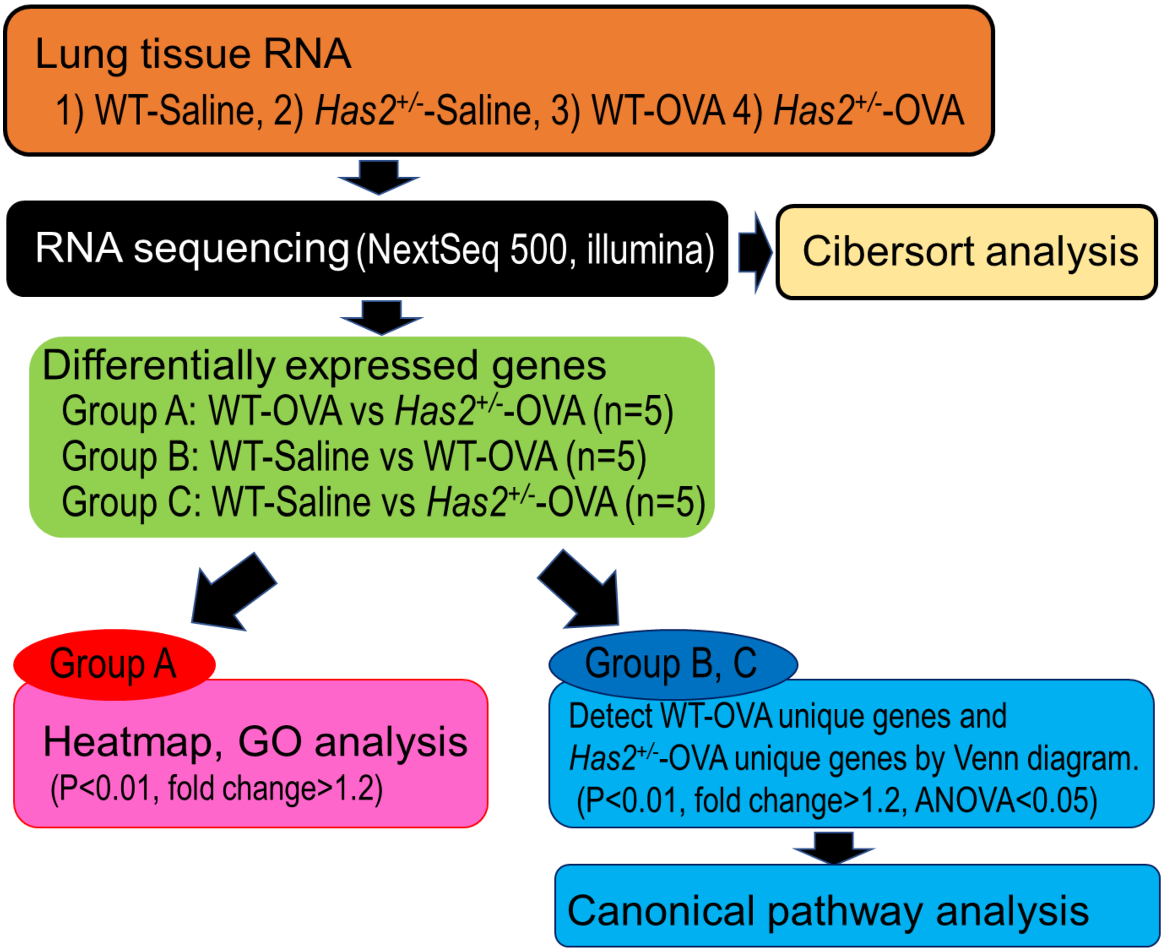

Supplementary figure 1. Schematic workflow for the analysis of gene lists generated from the transcriptome profiling experiments. Gene lists represent gene products significantly upregulated and downregulated in the lungs under the following three conditions: (group A) WT-OVA mice compared with *Has2*$^{+/-}$-OVA mice, (group B) WT-saline mice compared with WT-OVA mice, and (group C) WT-saline mice compared with *Has2*$^{+/-}$-OVA mice. Lists of DEGs were prepared and analyzed by following software: Cibersort (https://cibersort.stanford.edu/), Morpheus (https://software.broadinstitute.org/morpheus/), Metascape (http://metascape.org), and ingenuity pathways analysis (IPA) software. GO, gene ontology.

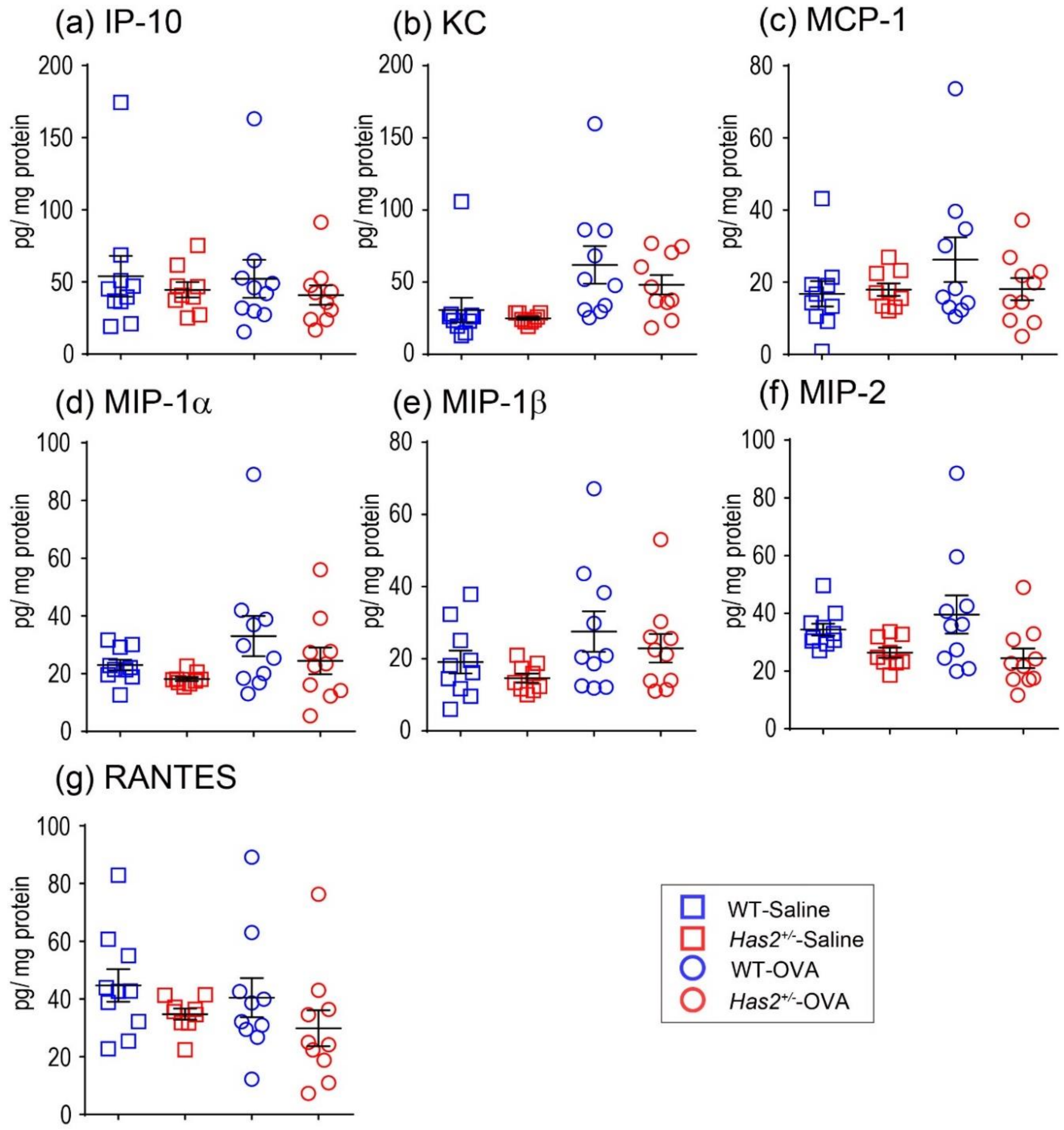

Supplementary figure 2. Effect of *Has2* attenuation on the inflammatory chemokine levels in lung homogenate. Protein adjusted levels of inflammatory chemokines in lung homogenates (n = 9–10). All samples are obtained 24 h after the final challenge with saline or OVA. Statistical significance was determined using Tukey's multiple comparison test.

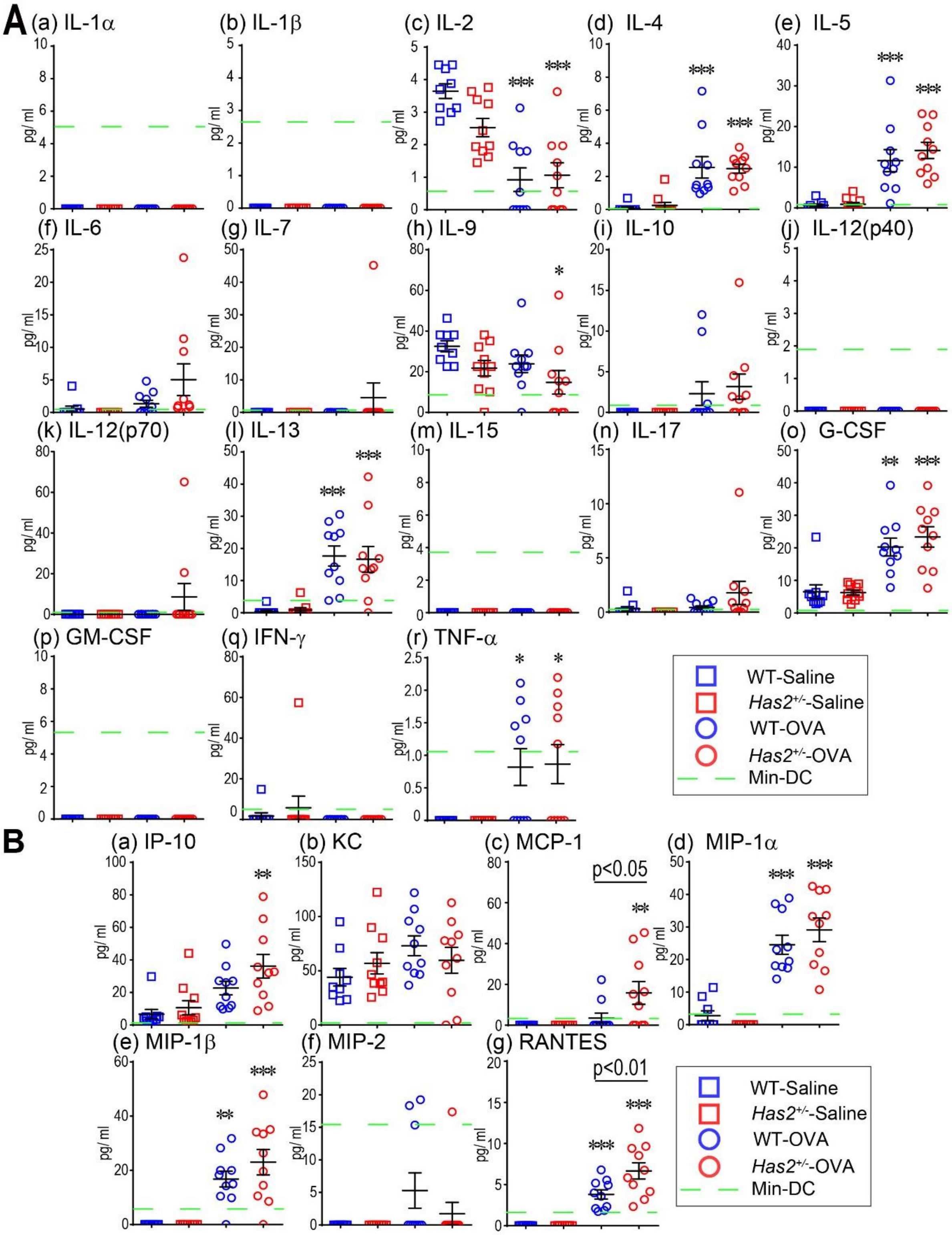
A
(a) IL-1α
(b) IL-1β
(c) IL-2
(d) IL-4
(e) IL-5
(f) IL-6
(g) IL-7
(h) IL-9
(i) IL-10
(j) IL-12(p40)
(k) IL-12(p70)
(l) IL-13
(m) IL-15
(n) IL-17
(o) G-CSF
(p) GM-CSF
(q) IFN-γ
(r) TNF-α
pg/ml
WT-Saline
Has2+/--Saline
WT-OVA
Has2+/--OVA
Min-DC
B
(a) IP-10
(b) KC
(c) MCP-1
p<0.05
(d) MIP-1α
(e) MIP-1β
(f) MIP-2
(g) RANTES
p<0.01
WT-Saline
Has2+/--Saline
WT-OVA
Has2+/--OVA
Min-DC

Supplementary figure 3. Effect of *Has2* attenuation on various cytokine and chemokine levels in BALF. (**A**) Levels of cytokines in BALF (n = 9–10). (**B**) Levels of chemokines in BALF (n = 9–10). All samples are obtained 24 h after the final challenge with saline or OVA. Statistical significance was determined using Tukey's multiple comparison test. *P < 0.05, **P < 0.01, and ***P < 0.001 relative to WT-saline mice. Horizontal bars indicate direct statistical comparisons between WT-OVA and *Has2*$^{+/-}$-OVA mice. *Dashed green lines* indicate the level of detection.

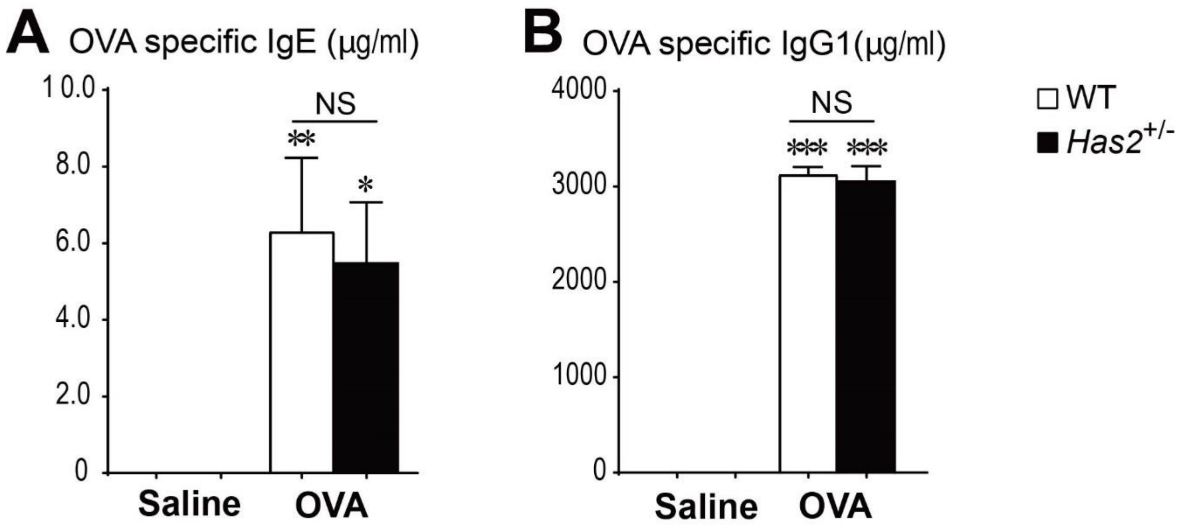

Supplementary figure 4. Effects of *Has2* attenuation on OVA-induced serological markers associated with allergic sensitization. Levels of total IgE (**A**) and OVA-specific IgE (**B**) in the serum were obtained 24 h after the final challenge with saline or OVA (n = 7). *P < 0.05; **P < 0.01; ***P < 0.001 relative to the WT-saline mice group as determined using Tukey's multiple comparison test. Horizontal bars indicate direct statistical comparisons between WT-OVA and *Has2*$^{+/-}$-OVA mice. NS, not significant.

Supplementary Table 1. Significant terms detected using canonical pathway analysis with IPA in WT-OVA unique differentially expressed genes.

Canonical Pathways	-log10(*p*-value)	z-score	Molecules
EIF2 Signaling	13.70	2.236	*AGO1, EIF1AX, EIF2AK3, EIF4G3, IGF1R, INSR, KRAS, RPL10A, RPL11, RPL12, RPL14, RPL17, RPL18, RPL19, RPL24, RPL28, RPL35, RPL37, RPL41, RPL6, RPL8, RPLP2, RPS10, RPS20, RPS23, RPS28, RPS3, RPS7, XIAP*
Axonal Guidance Signaling	4.50	NaN	*ADAMDEC1, ADAMTS3, ADAMTS4, ARHGEF12, ARPC2, BDNF, BMP4, CXCR4, FZD2, GNG11, HHIP, KRAS, MMP2, MYL3, NRP1, NRP2, NTNG2, PRKCZ, PRKD1, ROBO2, SLIT2, SMO, TUBA1A, TUBB1, WNT2B, WNT9A*
RhoA Signaling	4.07	-0.632	*ARHGAP5, ARHGEF12, ARPC2, CDC42EP3, IGF1R, MYL3, NRP2, PIP5K1C, SEPTIN2, SEPTIN8, SEPTIN9*
IL-4 Signaling	3.95	NaN	*HLA-A, HLA-DQB1, HLA-DRB5, INPP5J, INPP5K, KRAS, NR3C1, NR3C2, TYK2*
Regulation of eIF4 and p70S6K Signaling	3.70	NaN	*AGO1, EIF1AX, EIF4G3, KRAS, PPP2R3A, PRKCZ, RPS10, RPS20, RPS23, RPS28, RPS3, RPS7*
Epithelial Adherens Junction Signaling	3.24	NaN	*ACVR2A, ARPC2, CLINT1, KRAS, MYH6, MYL3, PARD3, SRC, TUBA1A, TUBB1, YES1*
Altered T Cell and B Cell Signaling in Rheumatoid Arthritis	3.09	NaN	*CD80, FASLG, HLA-A, HLA-DQB1, HLA-DRB5, TGFB1, TLR5, TRAF3*
Iron homeostasis signaling pathway	3.05	NaN	*ARNT, ATP6V0A4, ATP6V0C, ATP6V0E2, BMP4, BTRC, NUBP2, SLC39A14, TFRC, TYK2*
Factors Promoting Cardiogenesis in Vertebrates	3.00	NaN	*ACVR2A, BMP4, CCNE1, FZD2, PRKCZ, PRKD1, SMO, TGFB1*
Neuroinflammation Signaling Pathway	2.97	1	*ACVR2A, BDNF, CD80, FASLG, GRINA, HLA-A, HLA-DQB1, HLA-DRB5, IRAK1, MAPT, TGFB1, TICAM1, TLR5, TRAF3, TYK2, XIAP*
Unfolded protein response	2.86	NaN	*CEBPA, CEBPE, DNAJA2, EIF2AK3, OS9, UBXN4*
T Cell Exhaustion Signaling Pathway	2.74	1.134	*ACVR2A, BATF, CD80, HLA-A, HLA-DQB1, HLA-DRB5, IFNAR2, KRAS, PPP2R3A, TGFB1, TYK2*
14-3-3-mediated Signaling	2.68	-0.816	*KRAS, MAPT, PRKCZ, PRKD1, SRC, TUBA1A, TUBB1, VIM, YWHAB*
PCP pathway	2.67	-1.633	*FZD2, LGR4, PRICKLE1, SMO, WNT2B, WNT9A*
Role of Pattern Recognition Receptors in Recognition of Bacteria and Viruses	2.64	0.707	*C1QA, C1QB, C1QC, FASLG, NOD2, PRKCZ, PRKD1, TGFB1, TICAM1, TLR5*
mTOR Signaling	2.60	-1.342	*EIF4G3, INSR, KRAS, PPP2R3A, PRKCZ, PRKD1, RPS10, RPS20, RPS23, RPS28, RPS3, RPS7*
Virus Entry via Endocytic Pathways	2.58	NaN	*AP1B1, CLTA, HLA-A, KRAS, PRKCZ, PRKD1, SRC, TFRC*
Th2 Pathway	2.46	0.816	*ACVR2A, CD80, CXCR4, HLA-A, HLA-DQB1, HLA-DRB5, RUNX3, TGFB1, TYK2*

Graft-versus-Host Disease Signaling	2.41	NaN	*CD80, FASLG, HLA-A, HLA-DQB1, HLA-DRB5*
Role of Macrophages, Fibroblasts and Endothelial Cells in Rheumatoid Arthritis	2.39	NaN	*ADAMTS4, CEBPA, CEBPE, FZD2, IRAK1, KRAS, PRKCZ, PRKD1, SMO, SRC, TGFB1, TLR5, TRAF3, WNT2B, WNT9A*
Autoimmune Thyroid Disease Signaling	2.37	NaN	*CD80, FASLG, HLA-A, HLA-DQB1, HLA-DRB5*
Creatine-phosphate Biosynthesis	2.34	NaN	*CKB, CKMT1A/CKMT1B*
Th1 and Th2 Activation Pathway	2.30	NaN	*ACVR2A, CD80, CXCR4, HLA-A, HLA-DQB1, HLA-DRB5, NFIL3, RUNX3, TGFB1, TYK2*
IL-15 Production	2.28	NaN	*BMX, IGF1R, INSR, KIT, PRKCZ, SRC, TYK2, YES1*
Basal Cell Carcinoma Signaling	2.27	-2	*BMP4, FZD2, HHIP, SMO, WNT2B, WNT9A*
Phagosome Maturation	2.23	NaN	*ATP6V0A4, ATP6V0C, ATP6V0E2, DYNC2H1, HLA-A, HLA-DRB5, TUBA1A, TUBB1, VPS18*
Molecular Mechanisms of Cancer	2.20	NaN	*ADCY8, ARHGEF12, BMP4, CCNE1, FASLG, FZD2, KRAS, PRKCZ, PRKD1, RASGRF1, SMO, SRC, TGFB1, TYK2, WNT2B, WNT9A, XIAP*
Rapoport-Luebering Glycolytic Shunt	2.17	NaN	*BPGM, PGAM1*
Sumoylation Pathway	2.12	-0.447	*CEBPA, DNMT3A, FASLG, NR3C1, SUMO1, XIAP, ZEB1*
B Cell Development	2.11	NaN	*CD80, HLA-A, HLA-DQB1, HLA-DRB5*
Reelin Signaling in Neurons	2.11	NaN	*ARHGEF12, CNR2, MAPT, SRC, VLDLR, YES1*
Signaling by Rho Family GTPases	2.10	-0.333	*ARHGEF12, ARPC2, CDC42EP3, GNG11, MYL3, PARD3, PIP5K1C, PRKCZ, SEPTIN2, SEPTIN8, SEPTIN9, VIM*
p70S6K Signaling	2.10	-0.378	*EEF2, KRAS, MAPT, PPP2R3A, PRKCZ, PRKD1, SRC, YWHAB*
Acetyl-CoA Biosynthesis I (Pyruvate Dehydrogenase Complex)	2.03	NaN	*DBT, PDHA1*
Adipogenesis pathway	2.02	NaN	*ATG7, BMP4, BSCL2, CEBPA, FZD2, NR2F2, SMO, TGFB1*
STAT3 Pathway	2.00	-0.378	*CISH, IGF1R, IL17RD, INSR, KRAS, SRC, TGFB1, TYK2*
Colorectal Cancer Metastasis Signaling	1.97	-1.508	*ADCY8, FZD2, GNG11, KRAS, MMP2, SMO, SRC, TGFB1, TLR5, TYK2, WNT2B, WNT9A*
Regulation of the Epithelial-Mesenchymal Transition Pathway	1.97	NaN	*FGF23, FZD2, KRAS, MMP2, SMO, TGFB1, TYK2, WNT2B, WNT9A, ZEB1*
HER-2 Signaling in Breast Cancer	1.96	NaN	*CCNE1, KRAS, MMP2, PARD3, PRKCZ, PRKD1*
Breast Cancer Regulation by Stathmin1	1.87	NaN	*ADCY8, ARHGEF12, CCNE1, GNG11, KRAS, PPP2R3A, PRKCZ, PRKD1, TUBA1A, TUBB1*
Wnt/β-catenin Signaling	1.84	-1.667	*ACVR2A, BTRC, FZD2, PPP2R3A, SMO, SRC, TGFB1, WNT2B, WNT9A*
Calcium-induced T Lymphocyte Apoptosis	1.83	0.447	*HLA-A, HLA-DQB1, HLA-DRB5, PRKCZ, PRKD1*

Mitotic Roles of Polo-Like Kinase	1.83	0	*ANAPC1, FZR1, PPP2R3A, TGFB1, WEE1*
Role of NANOG in Mammalian Embryonic Stem Cell Pluripotency	1.78	NaN	*BMP4, FZD2, KRAS, SMO, TYK2, WNT2B, WNT9A*
NF-κB Signaling	1.74	-1.667	*BMP4, BTRC, IGF1R, INSR, IRAK1, KRAS, PRKCZ, TLR5, TRAF3*
Th1 Pathway	1.72	2.646	*CD80, HLA-A, HLA-DQB1, HLA-DRB5, NFIL3, RUNX3, TYK2*
Pentose Phosphate Pathway	1.72	NaN	*PGLS, TKT*
PTEN Signaling	1.67	1.134	*FASLG, IGF1R, INPP5J, INPP5K, INSR, KRAS, PRKCZ*
T Helper Cell Differentiation	1.66	NaN	*CD80, HLA-A, HLA-DQB1, HLA-DRB5, TGFB1*
Opioid Signaling Pathway	1.64	-0.905	*ADCY8, AP1B1, CLTA, GNG11, GRINA, KRAS, PRKCZ, PRKD1, RGS6, SRC, YES1*
Sperm Motility	1.61	NaN	*BMX, IGF1R, INSR, KIT, PRKCZ, PRKD1, PRKG2, SRC, TYK2, YES1*
UVC-Induced MAPK Signaling	1.60	-1	*KRAS, PRKCZ, PRKD1, SRC*
Xenobiotic Metabolism Signaling	1.59	NaN	*ALDH16A1, ARNT, EIF2AK3, FMO5, KRAS, MAOA, PPP2R3A, PRKCZ, PRKD1, SULT1D1, SUMO1, UGT1A7C*
Mouse Embryonic Stem Cell Pluripotency	1.57	-1.633	*BMP4, FZD2, KRAS, SMO, TYK2, XIAP*
RAR Activation	1.56	NaN	*ADCY8, DHRS7C, NR2F2, PRKCZ, PRKD1, RDH12, SMARCA2, SRC, TGFB1*
Clathrin-mediated Endocytosis Signaling	1.56	NaN	*AP1B1, ARPC2, CLTA, EPS15, FGF23, MYO6, PIP5K1C, SRC, TFRC*
Tec Kinase Signaling	1.53	-0.816	*BMX, FASLG, GNG11, PRKCZ, PRKD1, SRC, TYK2, YES1*
Glioblastoma Multiforme Signaling	1.52	-1.89	*CCNE1, FZD2, IGF1R, KRAS, SMO, SRC, WNT2B, WNT9A*
Gαs Signaling	1.52	0	*ADCY8, ADD3, ADORA2B, ADRB3, GNG11, SRC*
PD-1, PD-L1 cancer immunotherapy pathway	1.52	-2.236	*CD80, HLA-A, HLA-DQB1, HLA-DRB5, TGFB1, TYK2*
Human Embryonic Stem Cell Pluripotency	1.51	NaN	*BDNF, BMP4, FZD2, SMO, TGFB1, WNT2B, WNT9A*
Cdc42 Signaling	1.50	NaN	*ARPC2, HLA-A, HLA-DQB1, HLA-DRB5, MYL3, PARD3, PRKCZ, SRC*
Leukocyte Extravasation Signaling	1.49	0	*ARHGAP5, BMX, CXCR4, JAM2, MMP2, PRKCZ, PRKD1, SRC, TIMP1*
Gap Junction Signaling	1.49	NaN	*ADCY8, GRIK3, KRAS, PRKCZ, PRKD1, PRKG2, SRC, TUBA1A, TUBB1*
CXCR4 Signaling	1.49	-0.707	*ADCY8, CXCR4, GNG11, KRAS, MYL3, PRKCZ, PRKD1, SRC*
Cyclins and Cell Cycle Regulation	1.49	1	*BTRC, CCNE1, PPP2R3A, TGFB1, WEE1*

Ovarian Cancer Signaling	1.46	NaN	*FZD2, KRAS, MMP2, SMO, SRC, WNT2B, WNT9A*
Type I Diabetes Mellitus Signaling	1.45	NaN	*CD80, FASLG, HLA-A, HLA-DQB1, HLA-DRB5, IRAK1*
Phenylalanine Degradation IV (Mammalian, via Side Chain)	1.44	NaN	*GOT1, MAOA*
Aryl Hydrocarbon Receptor Signaling	1.41	0.816	*ALDH16A1, ARNT, CCNE1, ESR2, FASLG, SRC, TGFB1*
VEGF Family Ligand-Receptor Interactions	1.41	NaN	*KRAS, NRP1, NRP2, PRKCZ, PRKD1*
HIPPO signaling	1.41	1	*BTRC, PARD3, PPP2R3A, TP53BP2, YWHAB*
BMP signaling pathway	1.41	-2	*BMP4, FST, GREM1, KRAS, XIAP*
Nur77 Signaling in T Lymphocytes	1.39	NaN	*CD80, HLA-A, HLA-DQB1, HLA-DRB5*
Allograft Rejection Signaling	1.39	NaN	*CD80, FASLG, HLA-A, HLA-DQB1, HLA-DRB5*
PDGF Signaling	1.37	0.447	*INPP5J, INPP5K, KRAS, SRC, TYK2*
L-cysteine Degradation III	1.36	NaN	*GOT1*
Interferon Signaling	1.36	NaN	*IFIT1, IFNAR2, TYK2*
Synaptogenesis Signaling Pathway	1.35	-0.577	*ADCY8, AP1B1, ARPC2, BDNF, GRINA, KRAS, MAPT, RASGRF1, SRC, SYT11, VLDLR, YES1*
RhoGDI Signaling	1.34	0.378	*ARHGAP5, ARHGEF12, ARPC2, ESR2, GNG11, MYL3, PIP5K1C, SRC*
Chondroitin Sulfate Degradation (Metazoa)	1.33	NaN	*ARSB, HYAL1*
Complement System	1.33	NaN	*C1QA, C1QB, C1QC*
CTLA4 Signaling in Cytotoxic T Lymphocytes	1.32	NaN	*AP1B1, CD80, CLTA, HLA-A, PPP2R3A*
Acute Myeloid Leukemia Signaling	1.32	NaN	*CEBPA, KIT, KRAS, PIM2, RUNX1*

Supplementary Table 2. Significant terms detected using canonical pathway analysis with IPA in *Has2*$^{+/-}$-OVA unique differentially expressed genes.

Canonical Pathways	-log$_{10}$(p-value)	z-score	Molecules
Wnt/β-catenin Signaling	3.38	-0.378	*ACVR1, FZD5, FZD8, ILK, JUN, PPP2CB, SOX17, TLE1, WNT3A*
Neuroinflammation Signaling Pathway	3.25	0	*ACVR1, APH1A, APH1B, CASP1, IRAK2, JUN, MAPK6, NFE2L2, PPP3CA, SOD2, TLR1, TLR8*
Notch Signaling	2.85	NaN	*APH1A, APH1B, DLL1, MAML3*
TGF-β Signaling	2.82	-1	*ACVR1, JUN, MRAS, SMAD7, VDR, ZNF423*
Molecular Mechanisms of Cancer	2.74	NaN	*ADCY2, APH1A, APH1B, CRK, E2F8, FZD5, FZD8, GNA11, JUN, MRAS, RASGRP1, SMAD7, WNT3A*
Mouse Embryonic Stem Cell Pluripotency	2.64	-1.633	*FZD5, FZD8, ID2, ID3, MRAS, WNT3A*
Toll-like Receptor Signaling	2.53	NaN	*IRAK2, JUN, LBP, TLR1, TLR8*
IL-1 Signaling	2.19	NaN	*ADCY2, GNA11, IRAK2, JUN, MRAS*
Role of Macrophages, Fibroblasts and Endothelial Cells in Rheumatoid Arthritis	2.12	NaN	*CEBPD, FZD5, FZD8, IRAK2, JUN, MRAS, PPP3CA, TLR1, TLR8, WNT3A*
PCP pathway	2.10	-2	*FZD5, FZD8, JUN, WNT3A*
Human Embryonic Stem Cell Pluripotency	2.08	NaN	*ACVR1, FZD5, FZD8, MRAS, SMAD7, WNT3A*
Endothelin-1 Signaling	1.97	-0.378	*ADCY2, CASP1, GNA11, JUN, MAPK6, MRAS, PLB1*
ErbB4 Signaling	1.92	NaN	*APH1A, APH1B, MRAS, YAP1*
T Cell Receptor Signaling	1.92	NaN	*CARD11, JUN, MRAS, PPP3CA, RASGRP1*
Regulation of the Epithelial-Mesenchymal Transition Pathway	1.89	NaN	*APH1A, APH1B, FZD5, FZD8, ID2, MRAS, WNT3A*
Relaxin Signaling	1.88	-2	*ADCY2, GNA11, JUN, MRAS, NPR1, PDE8B*
Basal Cell Carcinoma Signaling	1.84	-1	*FZD5, FZD8, GLI3, WNT3A*
Colorectal Cancer Metastasis Signaling	1.76	-1.414	*ADCY2, FZD5, FZD8, JUN, MRAS, TLR1, TLR8, WNT3A*
iNOS Signaling	1.70	NaN	*IRAK2, JUN, LBP*
Cyclins and Cell Cycle Regulation	1.69	2	*CCNB1, CCNB2, E2F8, PPP2CB*
Osteoarthritis Pathway	1.68	-0.816	*ALPL, CASP1, FZD5, FZD8, GLI3, SMAD7, WNT3A*

Integrin Signaling	1.67	-1.633	*ARF2, CRK, ILK, ITGA2B, MPRIP, MRAS, TLN2*
1D-myo-inositol Hexakisphosphate Biosynthesis II (Mammalian)	1.66	NaN	*INPP5A, ITPKB*
D-myo-inositol (1,3,4)-trisphosphate Biosynthesis	1.66	NaN	*INPP5A, ITPKB*
BMP signaling pathway	1.62	NaN	*JUN, MRAS, SMAD7, ZNF423*
DNA damage-induced 14-3-3σ Signaling	1.61	NaN	*CCNB1, CCNB2*
Cardiac Hypertrophy Signaling (Enhanced)	1.61	-3.464	*ACVR1, ADCY2, FZD5, FZD8, GNA11, JUN, MKNK2, MRAS, NPR1, PDE8B, PPP3CA, WNT3A*
Choline Degradation I	1.59	NaN	*ALDH7A1*
Regulation of IL-2 Expression in Activated and Anergic T Lymphocytes	1.55	NaN	*CARD11, JUN, MRAS, PPP3CA*
Th2 Pathway	1.48	-1	*ACVR1, APH1A, APH1B, DLL1, JUN*
Superpathway of D-myo-inositol (1,4,5)-trisphosphate Metabolism	1.46	NaN	*INPP5A, ITPKB*
Unfolded protein response	1.45	NaN	*CEBPD, NFE2L2, PPP1R15A*
ATM Signaling	1.44	-1	*CCNB1, CCNB2, JUN, PPP2CB*
NRF2-mediated Oxidative Stress Response	1.43	NaN	*DNAJB1, DNAJB5, JUN, MRAS, NFE2L2, SOD2*
5-aminoimidazole Ribonucleotide Biosynthesis I	1.42	NaN	*PFAS*
ERK/MAPK Signaling	1.40	-0.816	*CRK, DUSP6, MKNK2, MRAS, PPP2CB, TLN2*
Wnt/Ca^{+} pathway	1.34	NaN	*FZD5, FZD8, PPP3CA*
